# Effectiveness of Platelet‐Rich Fibrin for Temporomandibular Disorders Management: A Systematic and Meta‐Analysis

**DOI:** 10.1111/odi.70089

**Published:** 2025-09-07

**Authors:** Júlia Silva Gomes de Araújo, Wellington Luiz de Oliveira da Rosa, Luiz Augusto Duarte Meirelles, Mateus Gaya dos Santos, Noéli Boscato

**Affiliations:** ^1^ Graduate Program in Dentistry Federal University of Pelotas Pelotas Brazil; ^2^ Graduate Program in Dentistry Oral and Maxillofacial Surgery Faculty Clinic, The Ohio State University Columbus USA

**Keywords:** arthrocentesis, arthroscopy, platelet‐rich fibrin, temporomandibular disorder, temporomandibular joint

## Abstract

**Objective:**

This study systematically reviewed the effect of intra‐articular injection (IAI) of platelet‐rich fibrin (i‐PRF), alone or combined with arthrocentesis or arthroscopy, in managing painful articular temporomandibular disorders (TMD) and improving maximum mouth opening (MMO).

**Materials and Methods:**

A systematic literature search was conducted in five databases, identifying randomized and nonrandomized clinical trials focusing on minimally invasive TMJ interventions using i‐PRF. Meta‐analyses were performed with a random‐effects model for studies reporting similar outcomes, visual analog scale (VAS) scores for pain, and MMO values.

**Results:**

Out of 332 studies identified, 13 met the inclusion criteria for meta‐analysis. Within‐group comparisons revealed significant pain reduction at 3–6 and 8–12 months for i‐PRF alone or combined with arthrocentesis or arthroscopy. MMO values significantly increased using i‐PRF alone at 8–12 months, combined with arthrocentesis or arthroscopy at 3–6 and 8–12 months, with no significant increase using i‐PRF alone at 3–6 months. Between‐group analyses demonstrated that i‐PRF combined with arthrocentesis significantly reduced VAS scores and increased MMO values compared to arthrocentesis alone at 3–6 and 8–12 months.

**Conclusion:**

Using i‐PRF alone or combined with arthrocentesis or arthroscopy is effective in pain reduction and MMO increase.

## 1 Introduction

The temporomandibular joint (TMJ) is a complex joint (Granados 1979). The TMJ disc must remain in the correct position during functional movements and retain its normal shape. When this balance is disrupted, temporomandibular disorder (TMD) arises (Chang et al. 2018) a condition that affects the TMJs, the masticatory muscles, surrounding bony structures, and soft tissues, or a combination of these components (Durham et al. 2015). Signs and symptoms of TMDs include TMJ sounds and pain, headaches, facial and neck pain, limited mandibular range of motion, and reduced maximum mouth opening (MMO) (Durham et al. 2015).

The management of articular TMDs includes noninvasive, minimally invasive, and invasive surgical interventions (Dasukil et al. 2021). Noninvasive procedures are the first‐choice therapy; however, if they fail to sufficiently reduce painful symptoms, minimally invasive interventions, such as arthrocentesis and arthroscopy, either alone or combined with intra‐articular injection (IAI), are considered before progressing to invasive surgeries. Studies suggested that combining IAI with arthrocentesis offers greater effectiveness than only arthrocentesis in managing internal temporomandibular derangement (Ghoneim et al. 2022). In this context, platelet‐rich fibrin (i‐PRF) has emerged as a second‐generation platelet concentrate. Unlike platelet‐rich plasma (PRP), a first‐generation concentrate derived from blood centrifugation, i‐PRF is generated without anticoagulants, allowing one to produce blood‐derived matrices that may promote tissue regeneration and healing (Choukroun and Ghanaati 2018; Marx et al. 1998).

Arthrocentesis is a minimally invasive surgical intervention used for managing painful articular TMDs and is often regarded as a first‐line treatment for patients who do not respond to conservative therapies (Toameh et al. 2019). This procedure alleviates negative intra‐articular pressure, releases adhered discs, restores mandibular movement, and removes damaged tissue and inflammatory pain mediators (Kılıç and Güngörmüş 2016; Singh et al. 2018). Arthroscopy is another minimally invasive intervention that needs special equipment that allows the visualization of joint cavities and tissues and provides additional benefits, including joint visualization, diagnostic capabilities, irrigation, biopsy, removal of adhesions, and the ability to address trauma in the lateral capsule (Moses et al. 1989; Nitzan and Dolwick 1991; Sanders 1986).

The combination of IAI using biological agents like i‐PRF with arthrocentesis has shown potential as a biosupplementation strategy, aiding in the regeneration of the TMJ's microarchitecture and providing long‐term relief from painful symptoms and functional limitation (Yüce and Kömerik 2020). While arthrocentesis alone has been reported to achieve an 83.5% success rate in symptom relief and MMO improvement, it does not necessarily address the underlying causes of painful TMDs (Nitzan 1994; Singh et al. 2018). Given the importance of systematic reviews in guiding clinical decision‐making, there is a need to synthesize and critically evaluate the available evidence on the use of i‐PRF, alone or in combination with arthrocentesis and arthroscopy, in managing articular TMDs (Xu et al. 2023). Previous reviews (Nemeth et al. 2024) have either not focused on the specific role of i‐PRF or have failed to comprehensively analyze all available randomized and nonrandomized trials. Thus, this systematic review aims to summarize the current scientific evidence on the effects of i‐PRF‐based IAI, alone or combined with arthrocentesis and arthroscopy, in reducing pain and improving MMO in individuals with articular TMDs.

## 2 Materials and Methods

The systematic review was carried out following the guidelines presented by the Cochrane Handbook for Systematic Reviews of Interventions and has been reported as per the four‐phase flow diagram recommended by the Preferred Reporting Items for Systematic Reviews and Meta‐Analyses (PRISMA) guidelines. The review protocol was registered in the OSF database (registration number x263w).

PICO framework was developed as follows: *Population*: symptomatic individuals diagnosed with osteoarthritis and/or internal derangement of TMJ based on RDC (Research Diagnostic Criteria for Temporomandibular Disorders), DC (Diagnostic Criteria for Temporomandibular Disorders) criteria, or Wilkes classification; *Intervention*: TMJ minimally invasive procedures (i.e., i‐PRF alone or i‐PRF combined with arthrocentesis or arthroscopy); *Comparison*: pre‐ and posttreatment within‐group comparisons: (a) i‐PRF alone, (b) i‐PRF + arthrocentesis, or (c) i‐PRF + arthroscopy, and between group‐comparisons: (a) arthrocentesis alone versus i‐PRF + arthrocentesis; Outcomes: VAS scores and MMO values. Review question: “Are minimally invasive procedures involving IAI of i‐PRF, alone or combined with arthrocentesis or arthroscopy, effective in reducing pain and increasing MMO in individuals diagnosed with articular TMD?”

This review included randomized clinical trials (RCTs) and nonrandomized clinical trials (non‐RCTs) assessing symptomatic patients diagnosed with articular TMD (i.e., osteoarthritis and/or internal derangement of TMJ) based on RDC, DC criteria, or Wilkes classification, and that using the IAI of i‐PRF alone, or i‐PRF combined with arthrocentesis or arthroscopy for the articular TMD management. Within‐group (i.e., before and after treatment) and between‐group comparisons (i.e., after treatment) were made. TMJ pain symptoms were assessed using VAS scale and MMO measures (i.e., distance between the incisal edges of the maxillary and mandibular central incisors). Literature reviews, case reports, case–control studies, systematic reviews, narrative reviews, and studies that did not provide information on MMO and pain assessment using VAS or have a follow‐up period of less than 1 month were excluded.

The search was carried out by two independent reviewers (J.A. and M.G.) using the following electronic databases: Medline/PubMed, Scopus, Embase (Elsevier), Web of Science, and Virtual Health Library: VHL (BIREME)—Portal Regional. The last search was performed on July 8, 2025, and no language or date restrictions were applied. The research strategy used is shown in the Supporting Information.

The search strategy was conducted across five databases following each syntax rule. The search protocol established for all databases is shown in Table S1. Additionally, the gray literature (ProQuest?) and the references of the studies included were also searched. No language restriction was applied.

The management of records and data throughout the review was conducted systematically using Rayyan Software by two independent reviewers to ensure accuracy and reproducibility. The studies searched were de‐duplicated using Rayyan (Intelligent Systematic Review) Software. The initial screening involved assessing the titles and abstracts of studies for their relevance. Studies that aligned with the inclusion criteria but lacked sufficient information in the title or abstract for a definitive decision were moved forward for full‐text review. In cases where duplicate studies or overlapping samples were identified, preference was given to the version with either a longer follow‐up duration or the most recent publication. After full‐text analysis, those that fulfilled the eligibility requirements were included in the data extraction stage. Additionally, reference lists of all qualifying studies were manually reviewed to minimize the risk of overlooking relevant publications. Any disagreements about study eligibility were resolved through discussion and consensus by a third reviewer (N.B.).

Data extraction was carried out using a standardized template in Microsoft Excel 2013 (Microsoft Corporation, Redmond, WA, USA), capturing the following information: study authors, publication year, study characteristics (including design, country of origin, inclusion criteria, sample size, and follow‐up duration), TMD diagnosis, TMJ intervention details, maximum mouth opening (MMO) measurements, visual analog scale (VAS) scores, the substance used during arthrocentesis, volume of i‐PRF (in mL), and the number of intra‐articular injections (IAIs) of i‐PRF. When data were incomplete or unpublished, the corresponding study authors were contacted via email.

### 2.1 Meta‐Analyses

Meta‐analyses were performed using Review Manager version 5.2 (The Nordic Cochrane Center, The Cochrane Collaboration, Copenhagen, Denmark) for studies that shared comparable control groups and reported identical outcome variables (VAS and MMO). A random‐effects model was applied for the overall analysis. Statistical significance was defined as a *p* value less than 0.05. Variability in treatment effects across studies was evaluated using Cochran's Q test along with the I^2^ statistic to assess heterogeneity.

Combined effect estimates for mean differences in VAS and MMO, along with their standard deviations (SD), were computed both within groups (pre‐ and posttreatment) and between groups following different interventions. The data were categorized based on follow‐up periods: From baseline up to 6 months, and from 8 to 12 months. Publication bias was assessed using funnel plots generated in RevMan for the outcomes of visual analog scale (VAS) and maximum mouth opening (MMO).

Two reviewers independently evaluated the risk of bias in each study. For randomized clinical trials, the RoB 2 tool was utilized, and studies were categorized as having “low risk,” “some concerns,” or “high risk” of bias. For nonrandomized clinical trials, the ROBINS‐I tool was applied, classifying studies into “low,” “moderate,” “serious,” or “critical” risk levels.

### 2.2 Data Synthesis

The quantitative analysis focused exclusively on VAS and MMO data, while outcomes presented as scores, percentages, or descriptive results were included in the qualitative synthesis. Sensitivity analyses were conducted to assess the robustness of pooled estimates, particularly after excluding non‐RCTs or studies identified as having a high risk of bias. Additional sensitivity assessments accounted for missing posttreatment data, interpreting these as either treatment success or failure scenarios.

To evaluate the evidence quality of the studies for Confidence in Cumulative Evidence, the GRADE (Grading of Recommendations, Assessment, Development, and Evaluations) method was used in Gradepro GDT (GRADEpro Guideline Development Tool [Software] McMaster University and Evidence Prime, 2022).

## 3 Results

**FIGURE 1:**
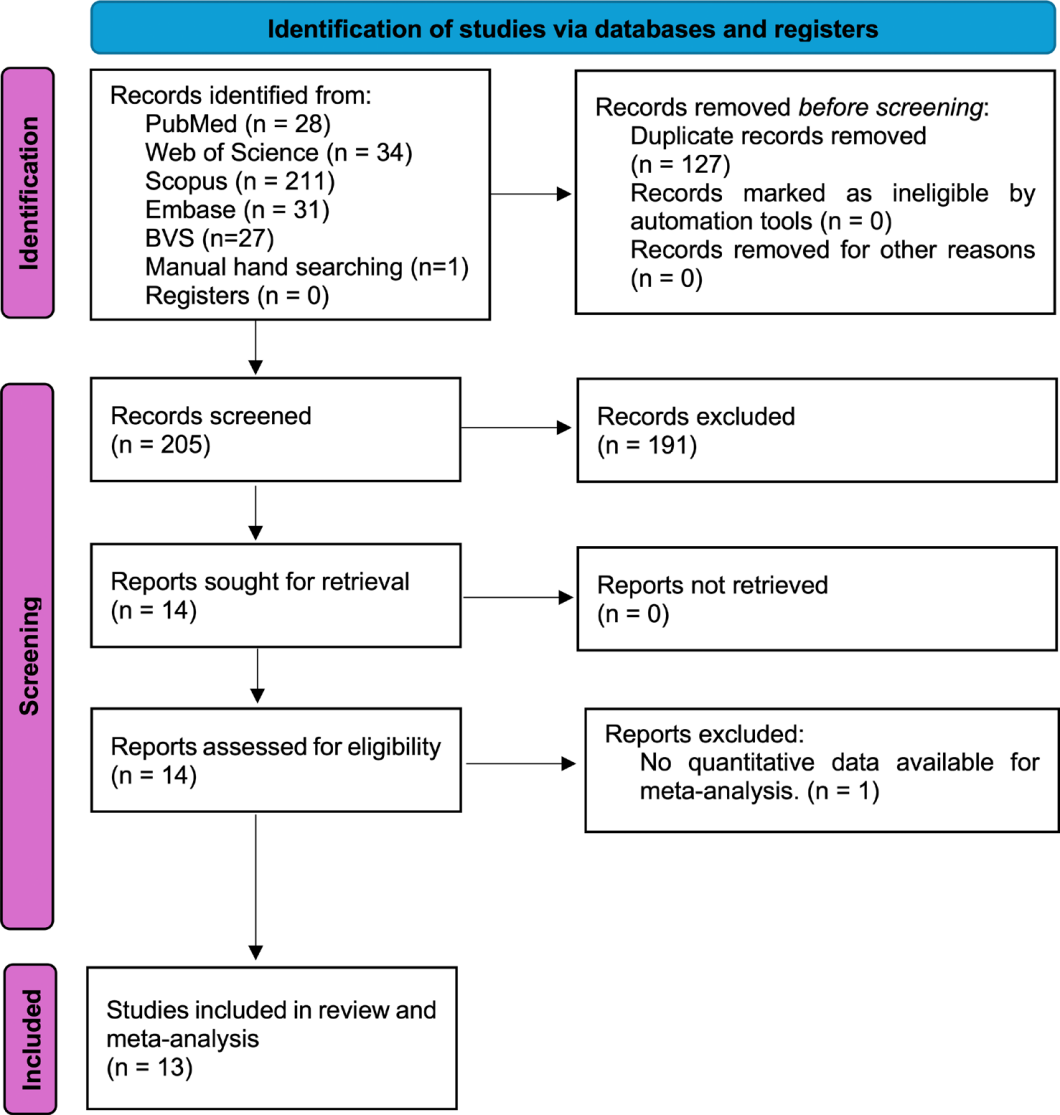
Flowchart of the systematic review.

The search initially identified 332 studies, with 127 duplicates subsequently removed (Figure 1). The remaining 205 studies had their titles, abstracts, and keywords screened, resulting in the exclusion of 191 articles that did not meet the eligibility criteria. Full‐text assessments were conducted on the remaining 14 studies, leading to the exclusion of one additional study due to insufficient data. All included studies mentioned ethical compliance approval.

The final selection comprised 13 studies that met all eligibility requirements, of which five were RCTs (two retrospectives, two prospective, and one descriptive observational study with retrospective case series) (Albilia et al. 2020; Bera and Tiwari 2022; Ghoneim et al. 2022; González et al. 2021; Işık et al. 2022; Işık et al. 2023; Karadayi and Gursoytrak 2021; Sharma et al. 2023; Torul et al. 2021; Yüce and Kömerik 2020). Only one RCT included in this review exhibited a high risk of bias, primarily due to the lack of blinding, as both assessors and participants were aware of the intervention. In contrast, the other four RCTs presented a low risk of bias (Figure S1a). Conversely, most of the non‐RCTs included in this review demonstrated a serious risk of bias, stemming from issues such as a lack of blinding, potential confounding effects of the intervention, differences in the initiation of follow‐up, and unbalanced comparisons between experimental groups (Figure S1b).

The funnel plot for the VAS outcome (Figure S2a) demonstrated an asymmetric distribution, with two studies on one side and four on the opposite side of the mean effect, suggesting potential publication bias and small‐study effects. Similarly, the funnel plot for the MMO outcome (Figure S2b) showed asymmetry, with four studies distributed on one side, two on the other, and one study near the center, indicating possible publication bias or heterogeneity among the included studies.

The studies included in the review were published between 2018 and 2025, and all 13 were incorporated into the meta‐analysis. Details regarding the study populations, surgical interventions performed, evaluated outcomes, and key findings are presented in Tables S2 and S3. All studies indicated that minimally invasive surgical procedures for TMD were performed only after the failure of initial conservative and nonsurgical treatments. According to the GRADE evaluation, the overall quality of evidence was generally rated as moderate (Table S4).

**FIGURE 2:**
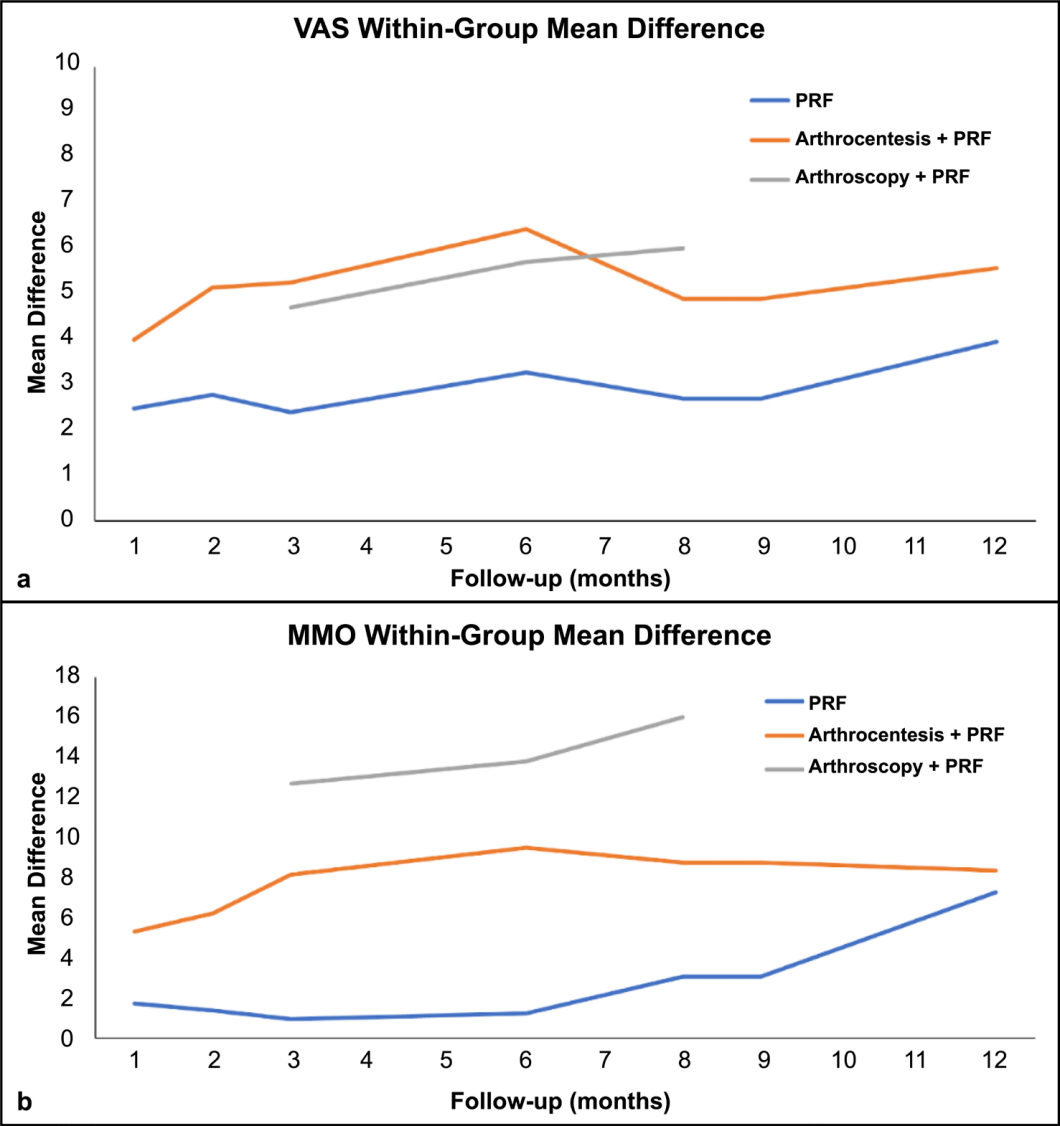
(a) Mean difference of within‐group comparisons graphic using the visual analog scale (VAS) in a 12‐month follow‐up; (b) Mean difference of within‐group comparisons graphic using the maximal mouth opening (MMO) in a 12‐month follow‐up.

The mean difference of within‐group comparisons at 12‐month follow‐up is shown in Figures 2a and 2b. The use of i‐PRF alone yielded a lower mean difference than i‐PRF combined with arthrocentesis or arthroscopy therapy. The i‐PRF combined with arthroscopy showed higher MMO mean difference values than i‐PRF combined with arthrocentesis (Figure 2b).

### 3.1 VAS and MMO Outcomes

**FIGURE 3:**
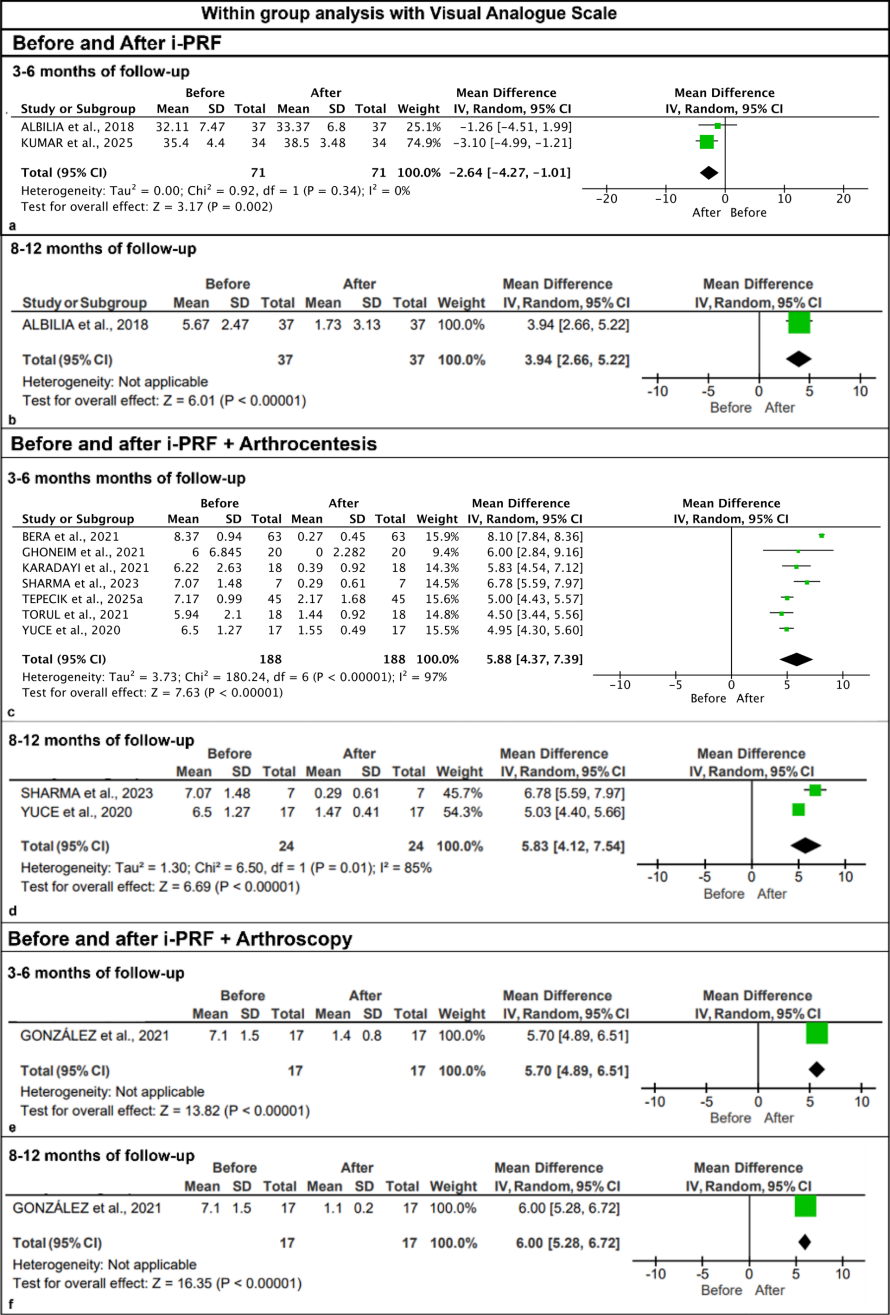
Meta‐analysis of within‐group analysis with the visual analog scale (VAS): (a,b) comparison before and after i‐PRF in a follow‐up of 3–6 months and 8–12 months, respectively, showing significant reduction in pain VAS scores after the intervention; (c,d) comparison before and after i‐PRF + arthrocentesis in a follow‐up of 3–6 months and 8–12 months, respectively, showing significant reduction in pain VAS scores after the intervention; (e,f) comparison before and after i‐PRF + arthroscopy in a follow‐up of 3–6 months and 8–12 months, respectively, showing significant reduction in pain VAS scores after the intervention.

**FIGURE 4:**
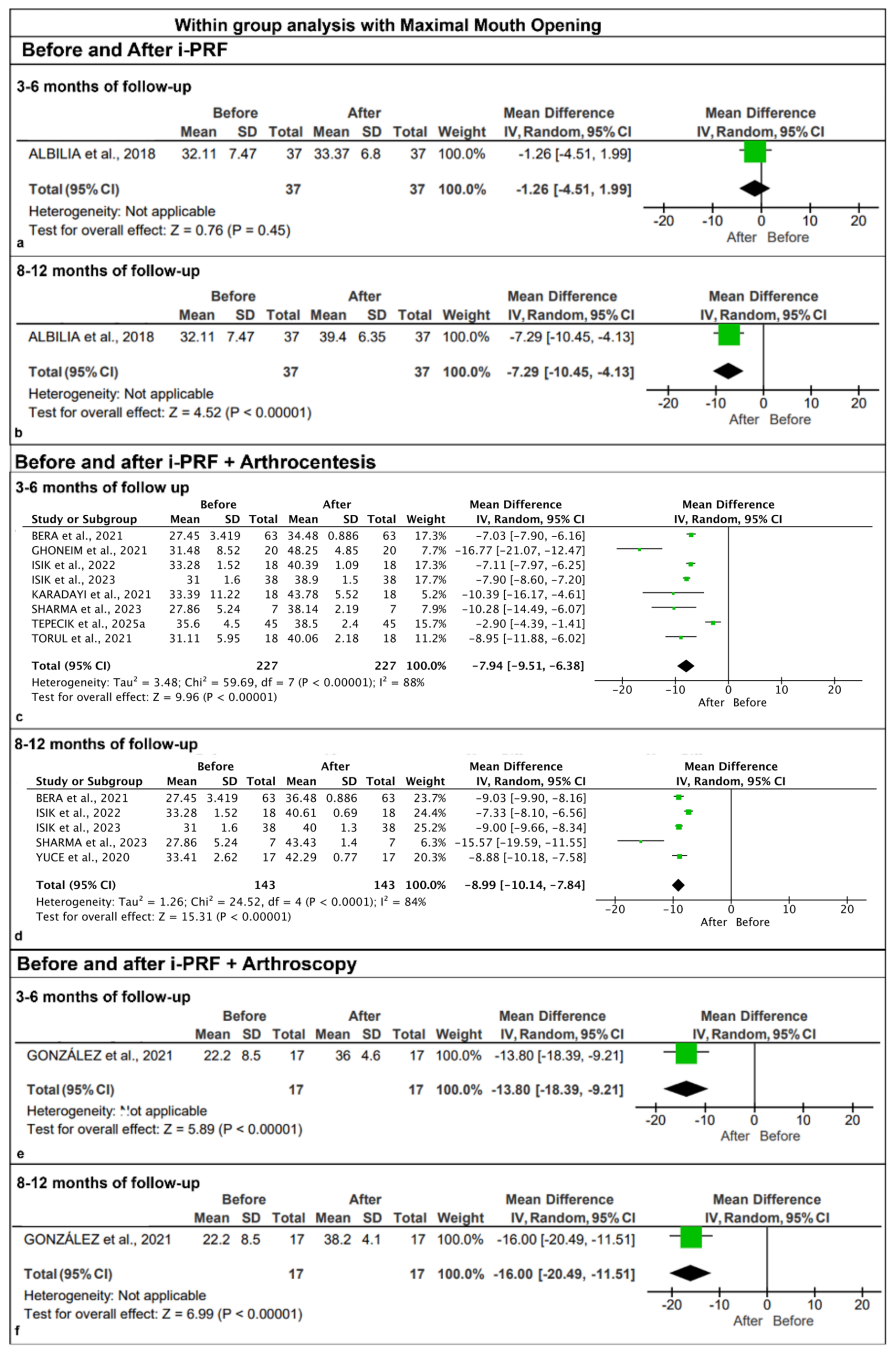
Meta‐analysis of within‐group analysis with the maximal mouth opening (MMO): (a,b) comparison before and after i‐PRF in a follow‐up of 3–6 months and 8–12 months, respectively, showing significantly favorable MMO results after the intervention; (c,d) comparison before and after i‐PRF + arthrocentesis in a follow‐up of 3–6 months and 8–12 months, respectively, showing significantly favorable MMO results after the intervention; (e,f) comparison before and after i‐PRF + arthroscopy in a follow‐up of 3–6 months and 8–12 months, respectively, showing significantly favorable MMO results after the intervention.

Ten studies evaluating VAS pain scores and MMO values were included. Overall, within‐group comparisons resulted in significantly lower VAS values (Figure 3) and increased MMO values (Figure 4). Before and after using the i‐PRF, after 3–6 months, a statistically significant difference was found in VAS scores (VAS, MD: −2.64 [−4.27, −1.01], *p* < 0.001) (Figure 3a), while no statistically significant difference was found in MMO values (MMO, MD: −1.26 [−4.51, 1.99], *p* = 0.45) (Figure 4a). Before and after using i‐PRF after 8–12 months, both VAS and MMO outcomes showed statistically significant differences (VAS, MD: 3.94 [2.66, 5.22], *p* < 0.00001, I^2^ = 0; MMO, MD: −7.29 [−10.45, −4.13], *p* < 0.00001) (Figures 3b and 4b).

Within‐groups comparisons using i‐PRF combined with arthrocentesis at 3–6 months (VAS, *p* < 0.00001, I^2^ = 97%, MD: 5.88; MMO, *p* < 0.00001, I^2^ = 88%, MD: −7.94) (Figures 3c and 4c), and also, at 8–12 months (VAS, *p* < 0.00001, I^2^ = 85%, MD: 5.83; MMO, *p* < 0.00001, I^2^ = 84%, MD: −8.99) resulted in VAS and MMO outcomes with a statistically significant difference (Figures 3d and 4d).

In the same way, using i‐PRF combined with arthroscopy at 3–6 months (VAS, *p* < 0.00001, MD: 5.70; MMO, *p* < 0.00001, MD: −13.80) (Figures 3e and 4e), and at 8–12 months (VAS, *p* < 0.00001, MD: 6.00; MMO, *p* < 0.00001, MD: −16.00) (Figures 3f and 4f), yielded VAS and MMO outcomes with statistically significant differences.

**FIGURE 5:**
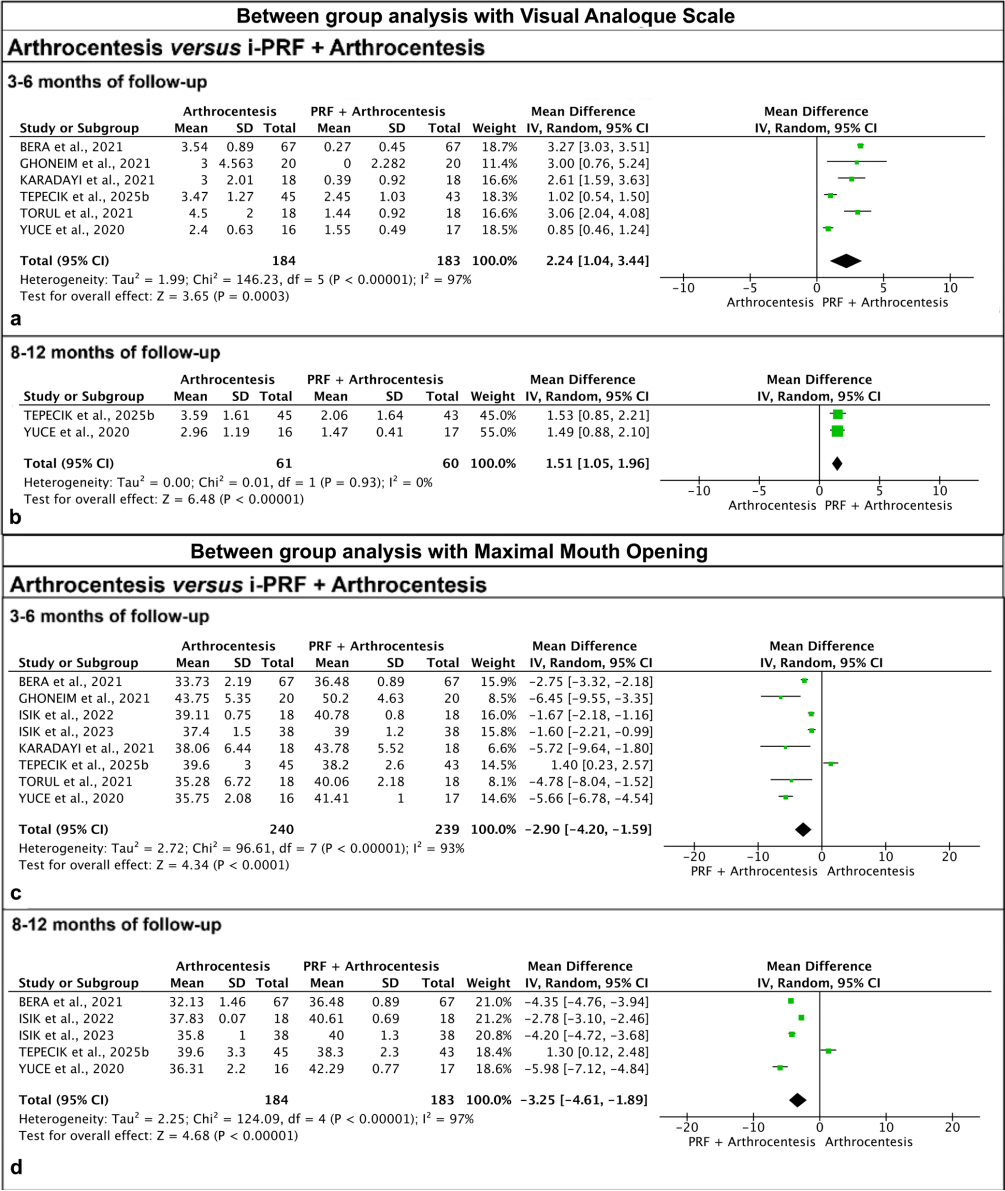
Meta‐analysis of between‐group analysis with the visual analog scale (VAS): (a,b) comparison between arthrocentesis versus i‐PRF + arthrocentesis in a follow‐up of 3‐6 months and 8–12 months, respectively, showing significant reduction in pain VAS scores after the intervention with i‐PRF. Meta‐analysis of between‐group analysis with the maximal mouth opening (MMO): (c,d) comparison between arthrocentesis versus i‐PRF + arthrocentesis in a follow‐up of 3–6 months and 8–12 months, respectively, showing significantly favorable MMO results after intervention with i‐PRF.

Between‐group comparisons showed that i‐PRF combined with arthrocentesis at 3–6 months showed significantly lower VAS scores (VAS, *p* = 0.0003, I^2^ = 97%, MD: 2.24), and at 8–12 months (VAS, *p* < 0.00001, MD: 1.51) than i‐PRF alone in both follow‐ups (Figure 5a,b). Increased MMO values were noted using i‐PRF combined with arthrocentesis (MMO, *p* < 0.00001, I^2^ = 93%, MD: −2.90) at 3–6 months and 8–12 months (MMO, *p* < 0.00001, MD: −3.25), with a statistically significant difference in both follow‐ups (Figure 5b,c).

## 4 Discussion

The results showed that IAI of i‐PRF, either alone or combined with arthrocentesis and arthroscopy, was effective in managing painful articular TMD and increasing MMO. However, the combination of i‐PRF with arthrocentesis and arthroscopy proved to be more effective. The combination of i‐PRF with arthrocentesis showed more consistent results due to higher grade evidence. In contrast, the use of i‐PRF alone and its combination with arthroscopy were supported by only one study each, providing lower grade evidence.

Across the studies, within‐group comparisons revealed significantly reduced VAS scores and increased MMO values in all follow‐ups, whether using i‐PRF alone or in combination with arthrocentesis or arthroscopy. However, combining i‐PRF with minimally invasive procedures led to greater improvements, demonstrating a more substantial reduction in VAS scores and a more pronounced increase in MMO. These findings align with previous studies suggesting that while i‐PRF alone offers benefits, its combination with adjunctive therapies such as arthrocentesis provides superior outcomes for pain management (Bera and Tiwari 2022). Additionally, this combination was more effective than arthrocentesis alone in managing internal TMJ derangement, resulting in enhanced functional outcomes and quality of life (Bera and Tiwari 2022). Studies using i‐PRF have also shown sustained analgesic effects and MMO improvements for 12 months. However, limitations in previous research include the lack of control groups using standard therapies or saline injections prior to i‐PRF.

Within‐group comparison of studies using i‐PRF combined with arthrocentesis was supported by a higher number of studies, showing consistent results and confidence intervals (CIs). Among the included studies, VAS analysis for 3–6 months of follow‐up involved 188 participants, with individual study sizes ranging from 7 to 63 participants, which may present limitations. For 8–12 months of follow‐up, the total sample size was 24 participants, ranging from 7 to 17. Small sample sizes (González et al. 2021; Sharma et al. 2023), especially those below 30, may reduce statistical power and reliability, while larger studies (Işık et al. 2023; Singh et al. 2018), closer to 63 participants, provide more generalizable results. For MMO analysis, the total sample size was 227 participants for 3–6 months and 143 participants for 8–12 months, with similar ranges. Despite these limitations, the evidence quality remains robust due to the consistent improvements in MMO and reductions in VAS scores.

Although these studies varied in design, some being RCTs and others non‐RCTs, and differed in protocol details, such as the number of injections administered (with some studies using multiple injections while others used a single injection), they still demonstrated similar results. The favorable results of i‐PRF in combination with arthrocentesis are likely because i‐PRF, generated through low‐speed centrifugation, has been shown to contain elevated levels of platelets, inflammatory cells, and growth factors, which are released in a sustained manner to promote cartilage regeneration (Torul et al. 2021).

One study included in the meta‐analysis evaluated arthroscopy combined with i‐PRF. Results showed an 84.6% reduction in clinical symptoms, with significant pain reduction (VAS) and a 41.7% increase in MMO (González et al. 2021). While this combination yielded higher MMO improvements compared to i‐PRF with arthrocentesis, it was supported by only one study (REF0) with a small sample size of 17 participants, leading to very low evidence based on GRADE criteria. Additionally, the study exhibited serious risks of bias due to inconsistent follow‐up initiation and unclear adjustments for selection bias. Further research is required to assess the positive effect of combining i‐PRF with arthroscopy, as no studies have directly compared this approach to arthroscopy alone.

Between‐group comparisons showed that i‐PRF combined with arthrocentesis yielded better outcomes than arthrocentesis alone, with significant reductions in VAS scores and increased MMO. All included studies showed similar CIs, with no inconsistency or imprecision per GRADE criteria. However, VAS evaluations and MMO results at 8–12 months displayed a serious risk of bias, while MMO at 3–6 months had a lower risk of bias due to the inclusion of more randomized studies. Probably, the i‐PRF used alone not improve MMO after 3–6 months, but only after 8–12 because a more reliable assessment of MMO is observed at 3–6 months posttreatment, as this period includes a higher proportion of RCTs, which minimize bias through rigorous study design, unlike the 8‐ to 12‐month follow‐up, where outcomes such as VAS and MMO showed a serious risk of bias due to the predominance of non‐RCTs (Albilia et al. 2020; Bera and Tiwari 2022; Ghoneim et al. 2022; Işık et al. 2022).

Arthrocentesis, being the least invasive and most straightforward procedure, effectively eliminates inflammatory mediators and lowers joint pressure (Bouchard et al. 2017). However, it does not alter the joint microenvironment (Cömert Kılıç and Güngörmüş 2016). When combined with i‐PRF, arthrocentesis shows improved long‐term effectiveness, as i‐PRF, processed through low‐speed centrifugation, evenly distributes platelets and leukocytes within a fibrin matrix, facilitating the gradual release of growth factors and inflammatory cells that aid in cartilage regeneration (Choukroun and Ghanaati 2018). The number of IAI may influence the treatment effectiveness. However, this contrasts with other included studies, which used a specific number of IAI for each patient, resulting in multiple injections, leading to nonstandardized treatment protocols and a higher risk of bias (Albilia et al. 2020; Yüce and Kömerik 2020). This issue could not be calculated in our meta‐analysis due to insufficient information available in the included studies. The number of IAI of i‐PRF used in the treatment of TMD varied significantly among the studies, reflecting differences in protocols and diagnostic criteria. One study (Albilia et al. 2020) applied multiple injections—averaging between 2.5 and 3.3 depending on Wilkes' stage—administered biweekly if patient improvement continued. Others (Bera and Tiwari 2022; Sharma et al. 2023) implemented six biweekly injections, reporting better outcomes in pain and MMO, respectively, over 12 and 9 months. Yüce and Kömerik (2020) used three weekly IAI, also reporting consistent improvements over a year. Indeed, [5, 20 23, 25] therapies even used a single IAI of i‐PRF and still observed significant pain reduction and improved mandibular motion. Two studies (Işık et al. 2022; Işık et al. 2023) also report progressive pain reduction and increased MMO maintained through 12 months using four weekly IAI without arthrocentesis. Besides, a recent study included in this review reported no additional benefit from administering three i‐PRF injections (1.5 mL) compared to a single injection (Tepecik et al. 2025). These findings suggest that while even a single IAI of i‐PRF can be effective, repeated applications may slightly enhance or prolong therapeutic outcomes depending on the case severity and protocol (Albilia et al. 2020; Bera and Tiwari 2022; Ghoneim et al. 2022; González et al. 2021; Işık et al. 2022; Işık et al. 2023; Karadayi and Gursoytrak 2021; Sharma et al. 2023; Yüce and Kömerik 2020).

The dose (volume, mL) of i‐PRF administered intra‐articularly may also have clinical significance, although current evidence remains inconclusive and somewhat heterogeneous, warranting further investigation. Across the studies analyzed, the volume of i‐PRF varied from 1 mL (Işık et al. 2022; Işık et al. 2023; Tepecik et al. 2025; Tepecik and Gedik 2025) to 2 mL (Albilia et al. 2020; González et al. 2021; Kumar et al. 2025; Sharma et al. 2023) per injection, with most studies reporting positive outcomes regardless of the exact volume used, single or multiple sessions, and weekly repetition. Meanwhile, favorable outcomes with both volumes suggest that therapeutic benefit may not be strictly dose‐dependent within this range (González et al. 2021). However, no study directly compared different i‐PRF volumes under controlled conditions, making it difficult to definitively establish a dose–response relationship. While IAI of i‐PRF volumes between 1 and 2 mL appears effective, the optimal dose remains undefined. It is plausible that a minimum threshold volume is required to coat the intra‐articular surfaces adequately, but exceeding this threshold may not proportionally enhance clinical effects. Higher volumes could theoretically increase intra‐articular pressure or discomfort, though no adverse effects were consistently reported (González et al. 2021). About this issue, further RCTs are necessary to determine the optimal injection protocol (Karadayi and Gursoytrak 2021; Sielski et al. 2023), investigating whether volume correlates with efficacy, especially regarding joint space, severity of TMD, and number of i‐PRF injections (Albilia et al. 2020; Bera and Tiwari 2022; Ghoneim et al. 2022; González et al. 2021; Işık et al. 2023; Sharma et al. 2023) to establish the best protocol.

Previous systematic review (Chęciński et al. 2023) showed that injections into the inferior TMJ compartment result in significantly better outcomes in pain reduction and an increase in MMO compared to the superior compartment. However, in the i‐PRF studies included in our review, injections were generally made into the superior compartment, with no studies explicitly targeting the inferior space—highlighting a gap and a potential area for future research.

The results of this systematic review comprise the limitations of the included studies, not only, but also including the sample size, absence of a control group, and lack of procedure standardization, which may guide further clinical trial designs comparing minimally invasive surgical procedures for the management of osteoarthritis and/or internal derangement of TMJ. Several limitations affect the strength of these conclusions, including the relatively short follow‐up periods in most studies, which limit the understanding of long‐term effectiveness and recurrence. The small sample sizes and potential publication bias reduce statistical power and increase the risk of bias, while heterogeneity and lack of standardization in i‐PRF injections may affect the biological properties and clinical outcomes of the procedure. However, although there were discrepancies in study designs, all included trials demonstrated a consistent trend indicating that i‐PRF, either alone or in combination with arthrocentesis or arthroscopy, may provide clinical benefits in reducing TMJ pain and enhancing MMO in patients with TMD; their direct application in clinical practice must be interpreted with caution. The findings of this systematic review, along with the limitations identified in the included studies, such as sample size, absence of control groups, and lack of procedural standardization, can inform the need for conducting further high‐quality RCTs comparing minimally invasive surgical techniques for managing osteoarthritis and/or internal derangement of the TMJ with standardized protocols and longer follow‐up periods before consistent clinical guidelines can be established based on strong scientific evidence. Comparing treatment methods with a definitive diagnosis is important, as failure to do this may render comparisons meaningless.

Despite variations in study designs, all the included studies revealed a consistent trend suggesting that i‐PRF, either alone or in combination with arthrocentesis or arthroscopy, may be beneficial in reducing TMJ pain and improving MMO. Future research should prioritize comparing treatment approaches with a definitive diagnosis, as failure to do so could undermine the validity of cross‐study comparisons.

## 5 Conclusion

Based on limited available evidence, procedures using i‐PRF, whether alone or combined with arthrocentesis or arthroscopy, have shown promise in decreasing TMJ pain (lower VAS scores) and increasing MMO (higher MMO values). The findings also suggest that the combination of i‐PRF with arthrocentesis is the most effective approach for pain reduction and functional improvement in TMJ disorders.

## Author Contributions

**Júlia Silva Gomes de Araújo:** writing – original draft, investigation, methodology, writing – review and editing, data curation, formal analysis. **Wellington Luiz de Oliveira da Rosa:** writing – review and editing, methodology, supervision, data curation, investigation. **Luiz Augusto Duarte Meirelles:** writing – review and editing, investigation. **Mateus Gaya dos Santos:** investigation, writing – review and editing. **Noéli Boscato:** conceptualization, investigation, writing – original draft, writing – review and editing, methodology, data curation, supervision.

## Ethics Statement

The authors have nothing to report.

## Consent

The authors have nothing to report.

## Conflicts of Interest

The authors declare no conflicts of interest.

## Supporting information


**Figure S1:** Risk of bias results using the (a) RoB‐2 tool for randomized clinical trials, and (b) the ROBINS‐I tool for nonrandomized clinical trials.
**Figure S2:** Funnel plots for publication bias assessment. (A) Funnel plot for visual analog scale (VAS) outcomes. (B) Funnel plot for maximum mouth opening (MMO) outcomes.
**Table S1:**. Search strategy for all databases.
**Table S2:** Characterization of the study sample.
**Table S3:** Description of the main results found in the studies, as well as TMD diagnostic methods.
**Table S4:** The overall quality of clinical recommendations for each of the main outcomes using the grades of recommendations, assessment, development, and evaluation (GRADE).
**Table S5:** PRISMA checklist.

## Data Availability

The data that support the findings of this study are available from the corresponding author upon reasonable request.
