# Supplementary material for: Effectiveness of Platelet‐Rich Fibrin for Temporomandibular Disorders Management: A Systematic and Meta‐Analysis
Source: Oral Dis. 2025 Sep 7;32(2):288–99. doi: 10.1111/odi.70089 (PMC13077014; doi:10.1111/odi.70089)
Supplement: Supplementary file 1 — Figure S1: Risk of bias results using the (a) RoB‐2 tool for randomized clinical trials, and (b) the ROBINS‐I tool for nonrandomized clinical trials. Figure S2: Funnel plots for publication bias assessment. (A) Funnel plot for visual analog scale (VAS) outcomes. (B) Funnel plot for maximum mouth opening (MMO) outcomes. Table S1:. Search strategy for all databases. Table S2: Characterization of the study sample. Table S3: Description of the main results found in the studies, as well as TMD diagnostic methods. Table S4: The overall quality of clinical recommendations for each of the main outcomes using the grades of recommendations, assessment, development, and evaluation (GRADE). Table S5: PRISMA checklist. [file ODI-32-288-s001.docx]

**Supplementary Materials**

**Supplementary Figure 1.** Risk of bias results using the (a) RoB-2 tool for Randomized Clinical Trials, and (b) the ROBINS-I tool for Non-randomized Clinical Trials

**Supplementary Figure 2.** Funnel plots for publication bias assessment. (A) Funnel plot for Visual Analog Scale (VAS) outcomes. (B) Funnel plot for Maximum Mouth Opening (MMO) outcomes.

**Supplementary Table 1**. Search strategy for all databases

|  | **Search Terms** |
| --- | --- |
|  | **Virtual Health Library: VHL (BIREME)** |
| #1 | ((Platelet-rich Fibrin) OR (Fibrin, Platelet-Rich) OR (Platelet Rich Fibrin) OR (L-PRF) OR (Leukocyte- and Platelet-Rich Fibrin) OR (Leukocyte and Platelet Rich Fibrin)) AND ((Temporomandibular joint) OR (Joint, Temporomandibular) OR (Joints, Temporomandibular) OR (Temporomandibular Joints) OR (TMJ)) AND ((Temporomandibular disorders) OR (TMD) OR (Temporomandibular joint) OR (Temporomandibular joint) OR (TMJ) OR (Temporomandibular joint disorders) OR (Temporomandibular Joint Disorders) OR (Temporomandibular joint syndrome) OR (Temporomandibular joint) OR (Temporomandibular articulation) OR (Joint temporomandibular) OR (Orofacial pain) OR (Facial pain) OR (Facial pain) OR (Temporomandibular Joint Dysfunction Syndrome) OR (Temporomandibular Joint Dysfunction Syndrome) OR (Disorders, Temporomandibular Joint) OR (Temporomandibular Joint Disorder) OR (TMJ Disorders) OR (TMJ Disorder) OR (Temporomandibular Disorders) OR (Temporomandibular Disorder) OR (Temporomandibular Joint Diseases) OR (Disease, Temporomandibular Joint) OR (TMJ Diseases) OR (TMJ Disease) OR (Temporomandibular Joints) OR (Temporomandibular Joint Disc) OR (Temporomandibular Joint Disc) OR (Temporomandibular Joint Discs) OR (Temporomandibular Articular Disc) OR (Temporomandibular Articular Discs) OR (Temporomandibular Articular Disk) OR (Temporomandibular Articular Disks) OR (Temporomandibular Joint Disk) OR (Temporomandibular Joint Disks) OR (Face Pain) OR (Pain, Facial) OR (Pain, Orofacial)) AND ((Arthrocentesis) OR (Arthrocenteses) OR (Arthrocentesis,Temporomandibular Joint) OR (Arthrocentesis,Temporomandibular Joints) OR (Joint, Arthrocentesis,Temporomandibular) OR (Joints, Arthrocentesis,Temporomandibular) OR (Arthrocentesis, TMJ) OR (Arthrocenteses, TMJ) OR (TMJ Arthrocenteses) OR (TMJ Arthrocentesis) OR (Temporomandibular Joint Aspiration) OR (Aspiration, Temporomandibular Joint) OR (Aspirations, Temporomandibular Joint) OR (Joint Aspiration, Temporomandibular) OR (Joint Aspirations, Temporomandibular) OR (Temporomandibular Joint Aspirations) OR (Temporomandibular Joint Arthrocentesis) OR (Arthrocenteses, Temporomandibular Joint) OR (Arthrocentesis, Temporomandibular Joint) OR (Joint Arthrocenteses, Temporomandibular) OR (Joint Arthrocentesis, Temporomandibular) OR (Temporomandibular Joint Arthrocenteses) OR (Aspiration,Temporomandibular Joint) OR (Aspiration,Temporomandibular Joints) OR (Joint, Aspiration,Temporomandibular) OR (Joints, Aspiration,Temporomandibular)) |
|  | **Pubmed** |
| #5 | #1 AND #2 AND #3 AND #4 |
| #4 | (Arthrocentesis) OR (Arthrocenteses) OR (Arthrocentesis,Temporomandibular Joint) OR (Arthrocentesis,Temporomandibular Joints) OR (Joint, Arthrocentesis,Temporomandibular) OR (Joints, Arthrocentesis,Temporomandibular) OR (Arthrocentesis, TMJ) OR (Arthrocenteses, TMJ) OR (TMJ Arthrocenteses) OR (TMJ Arthrocentesis) OR (Temporomandibular Joint Aspiration) OR (Aspiration, Temporomandibular Joint) OR (Aspirations, Temporomandibular Joint) OR (Joint Aspiration, Temporomandibular) OR (Joint Aspirations, Temporomandibular) OR (Temporomandibular Joint Aspirations) OR (Temporomandibular Joint Arthrocentesis) OR (Arthrocenteses, Temporomandibular Joint) OR (Arthrocentesis, Temporomandibular Joint) OR (Joint Arthrocenteses, Temporomandibular) OR (Joint Arthrocentesis, Temporomandibular) OR (Temporomandibular Joint Arthrocenteses) OR (Aspiration,Temporomandibular Joint) OR (Aspiration,Temporomandibular Joints) OR (Joint, Aspiration,Temporomandibular) OR (Joints, Aspiration,Temporomandibular) |
| #3 | (Temporomandibular disorders) OR (TMD) OR (Temporomandibular joint) OR (Temporomandibular joint) OR (TMJ) OR (Temporomandibular joint disorders) OR (Temporomandibular Joint Disorders) OR (Temporomandibular joint syndrome) OR (Temporomandibular joint) OR (Temporomandibular articulation) OR (Joint temporomandibular) OR (Orofacial pain) OR (Facial pain) OR (Facial pain) OR (Temporomandibular Joint Dysfunction Syndrome) OR (Temporomandibular Joint Dysfunction Syndrome) OR (Disorders, Temporomandibular Joint) OR (Temporomandibular Joint Disorder) OR (TMJ Disorders) OR (TMJ Disorder) OR (Temporomandibular Disorders) OR (Temporomandibular Disorder) OR (Temporomandibular Joint Diseases) OR (Disease, Temporomandibular Joint) OR (TMJ Diseases) OR (TMJ Disease) OR (Temporomandibular Joints) OR (Temporomandibular Joint Disc) OR (Temporomandibular Joint Disc) OR (Temporomandibular Joint Discs) OR (Temporomandibular Articular Disc) OR (Temporomandibular Articular Discs) OR (Temporomandibular Articular Disk) OR (Temporomandibular Articular Disks) OR (Temporomandibular Joint Disk) OR (Temporomandibular Joint Disks) OR (Face Pain) OR (Pain, Facial) OR (Pain, Orofacial) |
| #2 | (Temporomandibular joint) OR (Joint, Temporomandibular) OR (Joints,Temporomandibular) OR (Temporomandibular Joints) OR (TMJ) |
| #1 | (Platelet-rich Fibrin) OR (Fibrin, Platelet-Rich) OR (Platelet Rich Fibrin) OR (L-PRF) OR (Leukocyte- and Platelet-Rich Fibrin) OR (Leukocyte and Platelet Rich Fibrin) |
|  | **Web of Science** |
| #5 | #1 AND #2 AND #3 AND #4 |
| #4 | (Arthrocentesis) OR (Arthrocenteses) OR (Arthrocentesis,Temporomandibular Joint) OR (Arthrocentesis,Temporomandibular Joints) OR (Joint, Arthrocentesis,Temporomandibular) OR (Joints, Arthrocentesis,Temporomandibular) OR (Arthrocentesis, TMJ) OR (Arthrocenteses, TMJ) OR (TMJ Arthrocenteses) OR (TMJ Arthrocentesis) OR (Temporomandibular Joint Aspiration) OR (Aspiration, Temporomandibular Joint) OR (Aspirations, Temporomandibular Joint) OR (Joint Aspiration, Temporomandibular) OR (Joint Aspirations, Temporomandibular) OR (Temporomandibular Joint Aspirations) OR (Temporomandibular Joint Arthrocentesis) OR (Arthrocenteses, Temporomandibular Joint) OR (Arthrocentesis, Temporomandibular Joint) OR (Joint Arthrocenteses, Temporomandibular) OR (Joint Arthrocentesis, Temporomandibular) OR (Temporomandibular Joint Arthrocenteses) OR (Aspiration,Temporomandibular Joint) OR (Aspiration,Temporomandibular Joints) OR (Joint, Aspiration,Temporomandibular) OR (Joints, Aspiration,Temporomandibular) |
| #3 | (Temporomandibular disorders) OR (TMD) OR (Temporomandibular joint) OR (Temporomandibular joint) OR (TMJ) OR (Temporomandibular joint disorders) OR (Temporomandibular Joint Disorders) OR (Temporomandibular joint syndrome) OR (Temporomandibular joint) OR (Temporomandibular articulation) OR (Joint temporomandibular) OR (Orofacial pain) OR (Facial pain) OR (Facial pain) OR (Temporomandibular Joint Dysfunction Syndrome) OR (Temporomandibular Joint Dysfunction Syndrome) OR (Disorders, Temporomandibular Joint) OR (Temporomandibular Joint Disorder) OR (TMJ Disorders) OR (TMJ Disorder) OR (Temporomandibular Disorders) OR (Temporomandibular Disorder) OR (Temporomandibular Joint Diseases) OR (Disease, Temporomandibular Joint) OR (TMJ Diseases) OR (TMJ Disease) OR (Temporomandibular Joints) OR (Temporomandibular Joint Disc) OR (Temporomandibular Joint Disc) OR (Temporomandibular Joint Discs) OR (Temporomandibular Articular Disc) OR (Temporomandibular Articular Discs) OR (Temporomandibular Articular Disk) OR (Temporomandibular Articular Disks) OR (Temporomandibular Joint Disk) OR (Temporomandibular Joint Disks) OR (Face Pain) OR (Pain, Facial) OR (Pain, Orofacial) |
| #2 | (Temporomandibular joint) OR (Joint, Temporomandibular) OR (Joints,Temporomandibular) OR (Temporomandibular Joints) OR (TMJ) |
| #1 | (Platelet-rich Fibrin) OR (Fibrin, Platelet-Rich) OR (Platelet Rich Fibrin) OR (L-PRF) OR (Leukocyte- and Platelet-Rich Fibrin) OR (Leukocyte and Platelet Rich Fibrin) |
|  | **Scopus** |
| #5 | #1 AND #2 AND #3 AND #4 |
| #4 | (Arthrocentesis) OR (Arthrocenteses) OR (Arthrocentesis,Temporomandibular Joint) OR (Arthrocentesis,Temporomandibular Joints) OR (Joint, Arthrocentesis,Temporomandibular) OR (Joints, Arthrocentesis,Temporomandibular) OR (Arthrocentesis, TMJ) OR (Arthrocenteses, TMJ) OR (TMJ Arthrocenteses) OR (TMJ Arthrocentesis) OR (Temporomandibular Joint Aspiration) OR (Aspiration, Temporomandibular Joint) OR (Aspirations, Temporomandibular Joint) OR (Joint Aspiration, Temporomandibular) OR (Joint Aspirations, Temporomandibular) OR (Temporomandibular Joint Aspirations) OR (Temporomandibular Joint Arthrocentesis) OR (Arthrocenteses, Temporomandibular Joint) OR (Arthrocentesis, Temporomandibular Joint) OR (Joint Arthrocenteses, Temporomandibular) OR (Joint Arthrocentesis, Temporomandibular) OR (Temporomandibular Joint Arthrocenteses) OR (Aspiration,Temporomandibular Joint) OR (Aspiration,Temporomandibular Joints) OR (Joint, Aspiration,Temporomandibular) OR (Joints, Aspiration,Temporomandibular) |
| #3 | (Temporomandibular disorders) OR (TMD) OR (Temporomandibular joint) OR (Temporomandibular joint) OR (TMJ) OR (Temporomandibular joint disorders) OR (Temporomandibular Joint Disorders) OR (Temporomandibular joint syndrome) OR (Temporomandibular joint) OR (Temporomandibular articulation) OR (Joint temporomandibular) OR (Orofacial pain) OR (Facial pain) OR (Facial pain) OR (Temporomandibular Joint Dysfunction Syndrome) OR (Temporomandibular Joint Dysfunction Syndrome) OR (Disorders, Temporomandibular Joint) OR (Temporomandibular Joint Disorder) OR (TMJ Disorders) OR (TMJ Disorder) OR (Temporomandibular Disorders) OR (Temporomandibular Disorder) OR (Temporomandibular Joint Diseases) OR (Disease, Temporomandibular Joint) OR (TMJ Diseases) OR (TMJ Disease) OR (Temporomandibular Joints) OR (Temporomandibular Joint Disc) OR (Temporomandibular Joint Disc) OR (Temporomandibular Joint Discs) OR (Temporomandibular Articular Disc) OR (Temporomandibular Articular Discs) OR (Temporomandibular Articular Disk) OR (Temporomandibular Articular Disks) OR (Temporomandibular Joint Disk) OR (Temporomandibular Joint Disks) OR (Face Pain) OR (Pain, Facial) OR (Pain, Orofacial) |
| #2 | (Temporomandibular joint) OR (Joint, Temporomandibular) OR (Joints,Temporomandibular) OR (Temporomandibular Joints) OR (TMJ) |
| #1 | (Platelet-rich Fibrin) OR (Fibrin, Platelet-Rich) OR (Platelet Rich Fibrin) OR (L-PRF) OR (Leukocyte- and Platelet-Rich Fibrin) OR (Leukocyte and Platelet Rich Fibrin) |
|  | **Embase** |
| #5 | #1 AND #2 AND #3 AND #4 |
| #4 | (Arthrocentesis) OR (Arthrocenteses) OR (Arthrocentesis,Temporomandibular Joint) OR (Arthrocentesis,Temporomandibular Joints) OR (Joint, Arthrocentesis,Temporomandibular) OR (Joints, Arthrocentesis,Temporomandibular) OR (Arthrocentesis, TMJ) OR (Arthrocenteses, TMJ) OR (TMJ Arthrocenteses) OR (TMJ Arthrocentesis) OR (Temporomandibular Joint Aspiration) OR (Aspiration, Temporomandibular Joint) OR (Aspirations, Temporomandibular Joint) OR (Joint Aspiration, Temporomandibular) OR (Joint Aspirations, Temporomandibular) OR (Temporomandibular Joint Aspirations) OR (Temporomandibular Joint Arthrocentesis) OR (Arthrocenteses, Temporomandibular Joint) OR (Arthrocentesis, Temporomandibular Joint) OR (Joint Arthrocenteses, Temporomandibular) OR (Joint Arthrocentesis, Temporomandibular) OR (Temporomandibular Joint Arthrocenteses) OR (Aspiration,Temporomandibular Joint) OR (Aspiration,Temporomandibular Joints) OR (Joint, Aspiration,Temporomandibular) OR (Joints, Aspiration,Temporomandibular) |
| #3 | (Temporomandibular disorders) OR (TMD) OR (Temporomandibular joint) OR (Temporomandibular joint) OR (TMJ) OR (Temporomandibular joint disorders) OR (Temporomandibular Joint Disorders) OR (Temporomandibular joint syndrome) OR (Temporomandibular joint) OR (Temporomandibular articulation) OR (Joint temporomandibular) OR (Orofacial pain) OR (Facial pain) OR (Facial pain) OR (Temporomandibular Joint Dysfunction Syndrome) OR (Temporomandibular Joint Dysfunction Syndrome) OR (Disorders, Temporomandibular Joint) OR (Temporomandibular Joint Disorder) OR (TMJ Disorders) OR (TMJ Disorder) OR (Temporomandibular Disorders) OR (Temporomandibular Disorder) OR (Temporomandibular Joint Diseases) OR (Disease, Temporomandibular Joint) OR (TMJ Diseases) OR (TMJ Disease) OR (Temporomandibular Joints) OR (Temporomandibular Joint Disc) OR (Temporomandibular Joint Disc) OR (Temporomandibular Joint Discs) OR (Temporomandibular Articular Disc) OR (Temporomandibular Articular Discs) OR (Temporomandibular Articular Disk) OR (Temporomandibular Articular Disks) OR (Temporomandibular Joint Disk) OR (Temporomandibular Joint Disks) OR (Face Pain) OR (Pain, Facial) OR (Pain, Orofacial) |
| #2 | (Temporomandibular joint) OR (Joint, Temporomandibular) OR (Joints,Temporomandibular) OR (Temporomandibular Joints) OR (TMJ) |
| #1 | (Platelet-rich Fibrin) OR (Fibrin, Platelet-Rich) OR (Platelet Rich Fibrin) OR (L-PRF) OR (Leukocyte- and Platelet-Rich Fibrin) OR (Leukocyte and Platelet Rich Fibrin) |

**Supplementary Table 2.** Characterization of the study sample.

| **Study** | **Country** | **Inclusion criteria** | **Evaluated groups** | **Number of patients** | **Bilateral/Unilateral** | **Follow-up (months)** |
| --- | --- | --- | --- | --- | --- | --- |
| Albilia et al. 2018 | Canada | Patients with any degree of TMJ internal derangement and localized TMJ pain. | i-PRF alone | 37 | Unilateral or Bilateral | Preoperatively, 2, 4 and 8 weeks, 3, 6, 9 and 12 months |
| Bera; Tiwari, 2022 | India | Patients diagnosed both clinically and with imaging modalities (CT/MRI/CBCT) with osteoarthritis, treated with either arthrocentesis alone or in combination with i-PRF, minimum follow-up of 6 months, adequate data of treatment results at baseline and during follow-up for MMO and joint pain. | Arthrocentesis alone and i-PRF + arthrocentesis | 130 | Unilateral | MMO: 1, 3, 6 and 12 months; VAS: Pre-operatively and 15 days, 1, 3 and 6 months |
| Ghoneim et al. 2021 | Egypt | Patients suffering from TMJ internal derangement with reduction. All the included patients showed resistance to conservative treatment and recorded persistent restriction of mouth opening as well as TMJ pain and clicking. | Arthrocentesis alone and i-PRF + arthrocentesis | 40 | Unilateral | 1 week, 3 and 6 months |
| González et al. 2021 | Colombia | Patients over 18 years of age, with joint pain or previously diagnosed with Wilkes stage II and Wilkes stage III, and with a follow-up time of up to 8 months after the procedure. | i-PRF + arthroscopy | 17 | Unilateral | 3, 6, and 8 months |
| Ișik et al. 2022 | Turkey | Patients with Temporomandibular joint osteoarthritis (TMJ-osteoartrite) of one or two TMJs, maximum mouth opening less than 35 mm with passive stretch, and with deviation to the affected side, impeded protrusive and lateral movements, localized pain of the affected joint in palpation, chewing, and jaw movements, no response to conservative treatments, 18 years old, platelet counts of at least 150 000 mm, and willingness to participate in the study and to give informed consent. | Arthrocentesis alone and i-PRF + arthrocentesis | 36 | Unilateral or bilateral | 1, 2, 3, 6, and 12 months |
| Ișik et al. 2023 | Turkey | Patients with disc displacement without reduction (DDWoR), localized joint pain, limited mouth opening, impeded lateral and protrusive movements, no progress with conservative approaches over a 6-month period at least, and minimum age 18. | Arthrocentesis alone and i-PRF + arthrocentesis | 76 | Unilateral or bilateral | 1, 2, 3, 6, and 12 months |
| Karadayi; Gursoytrak, 2021 | Turkey | Patients with presence of unilateral internal temporomandibular disorder, presence of localized temporomandibular joint pain, scoring 3 and above in the Wilkes classification determined by magnetic resonance or computed tomography in addition to clinical evaluation. | Arthrocentesis alone and i-PRF + arthrocentesis | 36 | Unilateral | Preoperatively, and 10 and 30 days, and 3 months |
| Kumar et al., 2025 | India | Patients with TMJ internal derangement (Wilkes stages 3–5), unresponsive to at least 15 days of conservative treatment, and confirmed by clinical evaluation and MRI, were included. | i-PRF alone | 34 | Unilateral or bilateral | Preoperatively, and 10 and 30 days, and 3 months |
| Sharma et al., 2023 | India | Patients were classified under the Wilkes classification of internal derangement (stages I–V) based on clinical and radiographic evaluation and willingness to participate in the study. | PRP + arthrocentesis and i-PRF + arthrocentesis | 14 | Bilateral | Preoperative, and 1, 2, 3, 4, 5, 6 and 9 months |
| Tepecik et al., 2025a | Turkey | Patients included were females aged 18–75 with complete records, diagnosed with TMJ osteoarthritis based on DC/TMD and confirmed by CBCT, who had not received prior occlusal splint therapy, reported unilateral arthralgia with a VAS pain score ≥50 mm, and underwent arthrocentesis combined with i-PRF injections. | Single and multiple injections of i-PRF + arthrocentesis | 85 | Unilateral | Preoperative, and 1, 6 and 12 months |
| Tepecik et al., 2025b | Turkey | Patients included were females aged 18–75 with complete records, diagnosed with TMJ osteoarthritis based on DC/TMD and confirmed by CBCT, who had not received prior occlusal splint therapy, reported unilateral arthralgia with a VAS pain score ≥50 mm, and underwent arthrocentesis combined with i-PRF injections. | Arthrocentesis alone, i-PRF + Arthrocentesis and HA + Arthrocentesis | 127 | Unilateral | Preoperative, and 1, 6 and 12 months |
| Torul; Cezairli; Kahveci, 2021 | Turkey | Patients that showed Wilkes stage III aged between 18 and 65 years who had restricted mouth opening and TMJ pain and whose condition was refractory to conservative treatment were included in the study. | Arthrocentesis alone, i-PRF + Arthrocentesis and HA + Arthrocentesis | 54 | Unilateral | MMO: Preoperative, immediately after the procedure, and at 1 week, 1 and 3 months.  VAS: Preoperative, and at 1 week, 1 and 3 months. |
| Yuce; Komerik, 2020 | Turkey | Patients being a minimum age of 18 years, being diagnosed with any degree of TMJ internal derangement confirmed by magnetic resonance imaging with the presence of localized TMJ pain and limited mouth opening, with a minimum 12-month follow-up after undergoing treatment with arthrocentesis only (AO), arthrocentesis plus HA (AþHA), or arthrocentesis plus i-PRF (AþI-PRF). | Arthrocentesis only, HA + arthrocentesis, or i-PRF + arthrocentesis | 47 | Unilateral or bilateral | 2 weeks, and 1, 2, 3, 6, and 9 months |

| **Supplementary Table 3.** Description of the main results found in the studies, as well as TMD diagnostic methods. | | | | | | | | |
| --- | --- | --- | --- | --- | --- | --- | --- | --- |
| **Study** | **TMD Diagnostic Method** | **Evaluation Methods** | **Outcomes** | **Main results** | **Arthrocentesis (ml)** | **i-PRF (ml)** | **Number of injections** | **Protocol** |
| Albilia et al. 2018 | MRI according to the Wilkes classification | 1. VAS  2. MMO | 1. Pain  2. Mandibular range of motion | Exhibited a significant decrease in VAS scores and highest MMO values at the end of 12 months. | - | 2 ml | Multiple (according to the Wilkes’ classification were: stage II 3.16 ± 0.98; stage III 2.5 ± 0.70; stage IV 2.75 ± 1.13, and stage V 3.3 ± 1.56 | Every 2 weeks, provided the patient continues to report improvement |
| Bera; Tiwari, 2022 | Diagnostic Criteria for Temporomandibular Disorders (DC/TMD) | 1. VAS  2. MMO | 1. Pain  2. Mandibular range of motion | Exhibited a significant reduction in joint pain at the end of 6 months. At the end of 12 months, i-PRF + Arthrocentesis had significantly better mouth opening compared with Arthrocentesis alone. | 100 ml of Ringer's Lactate solution | 2 ml | 6 injections | Every 15 days for consecutive appointments |
| Ghoneim et al. 2021 | Clinical diagnosis of disc displacement with reduction confirmed by MRI | 1. VAS  2. MMO | 1. Pain  2. Mandibular range of motion  3. Clicking  4. Range of lateral mandibular excursions | Exhibited a significant decrease in VAS scores and significant increase in MMO scores compared to baseline. | 100 mL of 5% lactate solution | 1.5 ml | Single injection | - |
| González et al. 2021 | MRI according to the Wilkes classification | 1. VAS  2. MMO | 1. Pain  2.Mandibular range of motion  3. Facial nerve palsy | Exhibited a significant decrease in VAS scores and significant increase in MMO scores compared to baseline. | - | 1.5 and 2 ml | Single injection | - |
| Ișik et al. 2022 | Diagnostic Criteria for Temporomandibular Disorders (DC/TMD) | 1. VAS  2. MMO | 1. Pain  2. Mandibular range of motion  3. Lateral and protrusive movements | The pain levels were observed to decrease postoperatively at the 1^st^, 2^nd^, 3^rd^, and 6^th^ months, with these decreases in pain levels preserved through to the postoperative 12^th^ month, and the increases in MMO evaluated as pain values. | 200 mL of saline solution | 1 ml | 4 injections | Repeated on a weekly basis without an arthrocentesis procedure, with four consecutive i-PRF injections |
| Ișik et al. 2023 | Diagnostic Criteria for Temporomandibular Disorders (DC/TMD) | 1. VAS  2. MMO | 1. Pain  2. Mandibular range of motion  3. Lateral and protrusive movements | Significant decrease in pain levels and increase in the degree of MMO at the 1^s^t, 2^nd^, 3^th^, 6^th^, and 12^th^ months postoperatively. | 200 mL of saline solution | 1 ml | 4 injections | A repeat injection of i-PRF was made once a week without any arthrocentesis |
| Karadayi; Gursoytrak, 2021 | MRI or CT according to the Wilkes classification in addition to Helkimo index as a preliminary index | 1. VAS  2. MMO  3. Helkimo Clinical Dysfunction Index | 1. Pain  2.Mandibular range of motion  3. TMJ function  4. Muscle pain, TMJ area pain and mandibular movement | Exhibited a significant decrease in VAS scores and significant increase in MMO scores compared to baseline. | 100 mL of 5% lactate solution | 2 ml | Single injection | - |
| Kumar et al., 2025 | MRI according to the Wilkes classification | 1. MMO  2. Helkimo Clinical Dysfunction Index | 1. Mandibular range of motion  2. TMJ function  3. Muscle pain, TMJ area pain and mandibular movement | Exhibited a significant increase in MMO scores compared to baseline and improved Helkimo clinical dysfunction score | - | 2 ml | 3 injections | A repeat injection of i-PRF was made once a week |
| Sharma et al. 2023 | MRI according to the Wilkes classification | 1. VAS  2. MMO  3. More Rao Scale | 1. Pain  2. Mandibular range of motion  3. Lateral and protrusive movement  4. TMJ sounds  5. Disk position  6. Joint effusion | It was only showed a highly significant reduction in terms of VAS till 9 months of follow-up, when in terms of MMO, the improvement was found in the 3^rd^ month onward. | 200 ml ringer lactate solution | 2 ml | 6 injections | - |
| Tepecik et al., 2025a | Diagnostic Criteria for Temporomandibular Disorders (DC/TMD) and confirmed by CBCT | 1. VAS  2. MMO | 1. Pain  2. Mandibular range of motion | Exhibited a significant decrease in VAS scores and significant increase in MMO scores compared to baseline, with no benefit from increasing the frequency of i-PRF injections. | 100 mL of sodium chloride | 1.5 ml | 1 and 3 injections | A repeat injection of i-PRF was made once a week |
| Tepecik et al., 2025b | Diagnostic Criteria for Temporomandibular Disorders (DC/TMD) and confirmed by CBCT | 1. VAS  2. MMO | 1. Pain  2. Mandibular range of motion | All groups exhibited a significant decrease in VAS scores and significant increase in MMO scores compared to baseline, with no differences between groups. | 100ml of saline solution | 1.5 ml | 1 injection | - |
| Torul; Cezairli; Kahveci, 2021 | MRI according to the Wilkes classification | 1. VASr (pain at rest)  2. VASf (pain in function)  3. MMO | 1. Jaw pain at rest  2. Jaw pain in function  3. Mandibular range of motion | Significant improvements were observed in the two pain scores measured at 1 week, 1 month, and 3 months after the procedure when compared to baseline and significantly better MMO values were observed immediately after the procedure and at 1 week, 1 month, and 3 months postoperatively when compared baseline. | 100 ml of lactated Ringer’s solution | 1 ml | Single injection | - |
| Yuce; Komerik, 2020 | Research Diagnostic Criteria for Temporomandibular Disorders (RDC) | 1. VAS  2. MMO | 1. Pain  2. Mandibular range of motion | Statistically significant decreases in VAS scores and increases in MMO scoress were observed during the 12 months of follow-up. | 200 mL of lactated ringers solution | 2 ml | 3 injections of i-PRF at weekly intervals | - |

| **Supplementary Table 4.** The overall quality of clinical recommendations for each of the main outcomes using the Grades of Recommendations, Assessment, Development and Evaluation (GRADE). |
| --- |

**Question:** TMJ minimally invasive surgical procedures involving the intra-articular space comparing a) i-PRF alone, b) i-PRF + arthrocentesis, c) arthrocentesis *versus* i-PRF + arthrocentesis, and d) i-PRF + arthroscopy for symptomatic patients diagnosed with painful articular TMD such as osteoarthritis and/or internal derangement.

**Setting:** VAS

| **Certainty assessment** | | | | | | | **№ of patients** | | **Effect** | **Certainty** | | **Importance** | |
| --- | --- | --- | --- | --- | --- | --- | --- | --- | --- | --- | --- | --- | --- |
| **№ of studies** | **Study design** | **Risk of bias** | **Inconsistency** | **Indirectness** | **Imprecision** | **Other considerations** | **TMJ minimally invasive surgical procedures involving the intra-articular space** | **a) i-PRF alone, b) i-PRF +arthrocentesis, c) arthrocentesis versusi- PRF + arthrocentesis, and d) i-PRF + arthroscopy** | **Absolute (95% CI)** |  |  |  |  |
| **Before and After i-PRF (follow-up: range 3 months to 6 months)** | | | | | | | | | | | | | |
| 1 | non-randomised studies | serious^a^ | very serious^b^ | not serious | not serious | publication bias strongly suspected^b^ | 37 | 37 | MD **3.27 higher** (2.02 higher to 4.52 higher) | | ⨁◯◯◯ Very low^a,b^ | | CRITICAL |
| **Before and After i-PRF + Arthrocentesis (follow-up: range 3 months to 6 months)** | | | | | | | | | | | | | |
| 6 | randomised trials | not serious | not serious | not serious | serious^c^ | none | 143 | 143 | MD **6.04 higher** (4.39 higher to 7.7 higher) | | ⨁⨁⨁◯ Moderate^c^ | | CRITICAL |
| **Before and After i-PRF + Arthroscopy (follow-up: range 3 months to 3 months)** | | | | | | | | | | | | | |
| 1 | non-randomised studies | serious^d^ | very serious^e^ | not serious | serious^f^ | none | 17 | 17 | MD **5.7 higher** (4.89 higher to 6.51 higher) | | ⨁◯◯◯ Very low^d,e,f^ | | CRITICAL |
| **Arthrocentesis versus i-PRF + Arthrocentesis (follow-up: range 3 months to 6 months)** | | | | | | | | | | | | | |
| 5 | non-randomised studies | serious^g^ | not serious | not serious | not serious | none | 140 | 139 | MD **2.51 higher** (1.14 higher to 3.88 higher) | | ⨁⨁⨁◯ Moderate^g^ | | CRITICAL |
| **Before and After i-PRF (follow-up: range 8 months to 12 months)** | | | | | | | | | | | | | |
| 1 | non-randomised studies | serious^a^ | very serious^b^ | not serious | not serious | publication bias strongly suspected | 37 | 37 | MD **3.94 higher** (2.66 higher to 5.22 higher) | | ⨁◯◯◯ Very low^a,b^ | | CRITICAL |
| **Before and After i-PRF + Arthrocentesis (follow-up: range 8 months to 12 months)** | | | | | | | | | | | | | |
| 2 | non-randomised studies | serious^h^ | not serious | not serious | serious^i^ | none | 24 | 24 | MD **5.83 higher** (4.12 higher to 7.54 higher) | | ⨁⨁◯◯ Low^h,i^ | | CRITICAL |
| **Before and After i-PRF + Arthroscopy (follow-up: range 8 months to 12 months)** | | | | | | | | | | | | | |
| 1 | non-randomised studies | serious^d^ | very serious^e^ | not serious | not serious | publication bias strongly suspected | 17 | 17 | MD **6 higher** (5.28 higher to 6.72 higher) | | ⨁◯◯◯ Very low^d,e^ | | CRITICAL |
| **Arthrocentesis versus i-PRF + Arthrocentesis (follow-up: range 8 months to 12 months)** | | | | | | | | | | | | | |
| 1 | non-randomised studies | serious^j^ | not serious | not serious | not serious | none | 17 | 16 | MD **1.49 higher** (0.88 higher to 2.1 higher) | | ⨁⨁⨁◯ Moderate^j^ | | CRITICAL |

**CI:** confidence interval; **MD:** mean difference

#### Explanations

a. According to the ROBINS-I tool, in the Albilia study, there is potential for confounding of the intervention effect due to the absence of a control group. Based on the results, the author split participants into two groups: those who responded to the treatment and continued receiving i-PRF injections, and non-responders, for whom the i-PRF injections were discontinued.

b. Since this group consists of only one study (the Albilia study), there is a higher chance of inconsistency and a significant risk of publication bias.

c. The total sample size in the VAS analysis for the 3-6 months follow-up is 143 participants across studies, which provides a modest pool for analysis. However, individual study sizes (ranging from 7 to 63 participants) may present limitations. The 8-12 months follow-up has a total of 24 participants (ranging from 7 to 17 participants). Smaller sample sizes, especially those below 30, can reduce the statistical power of a study and increase the margin of error, potentially affecting the reliability of findings. In contrast, studies with larger samples, closer to 63 participants, are better suited to detect statistically significant effects and yield more generalizable results. However, most sample sizes in the "Before and After i-PRF + Arthrocentesis" group are small and may introduce serious imprecision.

d. According to the ROBINS-I tool, in the González study, the start of follow-up and the start of the intervention do not coincide for most participants, and it is not clear whether any adjustment techniques were used to correct for selection biases.

e. Since this group consists of only one study (the González study), there is a higher chance of inconsistency and a significant risk of publication bias.

f. The evaluation in this follow-up period is based on only one study, which also has a small sample size.

g. In the comparison of Arthrocentesis versus i-PRF + Arthrocentesis, there are five studies: three non-randomized and two randomized. The risk of bias was evaluated based on the worst-case scenario, which was deemed serious in the non-randomized studies, such as the Bera study. According to the ROBINS-I tool, the Bera study presented a serious risk of bias because it did not report whether the assessors and participants were aware of the intervention, and there was an imbalance in the number of participants between the control group and the experimental group.

h. In this follow-up period, there are two studies: one randomized trial and one non-randomized trial. The risk of bias was evaluated based on the non-randomized trial (Yuce study), which showed a serious risk of bias. This study reported an unbalanced comparison between the experimental groups, with the i-PRF group receiving multiple injections while the Hyaluronic Acid group received only a single injection. This discrepancy could affect the study's results. Furthermore, the start of follow-up and the start of the intervention do not coincide for most participants, as the procedures were performed between 2015 and 2018, and it is unclear whether the outcome assessors were aware of the intervention received by the participants.

i. The evaluation in this group is based on only two studies, both of which had small sample sizes.

j. This group has one study in these follow-ups, the Yuce study that presented a serious risk of bias due to the unbalanced comparison between the experimental groups. The i-PRF group received multiple injections, while the Hyaluronic Acid group received only one injection, which may have affected the study’s results. Additionally, the start of follow-up and the start of intervention do not coincide with most participants, as the procedures were carried out between 2015 and 2018. It is also not clear whether the outcome assessors were aware of which interventions the participants received.

**Absolute
(95% CI)**

**Question:** TMJ minimally invasive surgical procedures involving the intra-articular space comparing: a) i-PRF alone, b) i-PRF +arthrocentesis, c) arthrocentesis *versus* i-PRF + arthrocentesis, and d) i-PRF + arthroscopy for symptomatic patients diagnosed with painful articular TMD such as osteoarthritis and/or internal derangement.

**Setting:** MMO

**Abs**

| **Certainty assessment** | | | | | | | **№ of patients** | | **Effect** | **Certainty** | | **Importance** | |
| --- | --- | --- | --- | --- | --- | --- | --- | --- | --- | --- | --- | --- | --- |
| **№ of studies** | **Study design** | **Risk of bias** | **Inconsistency** | **Indirectness** | **Imprecision** | **Other considerations** | **TMJ minimally invasive surgical procedures involving the intra-articular space** | **a) i-PRF alone, b) i-PRF +arthrocentesis, c) arthrocentesis versus i-PRF + arthrocentesis, and d) i-PRF + arthroscopy** | **Absolute (95% CI)** |  | |  | |
| **Before and After i-PRF (follow-up: range 3 months to 6 months)** | | | | | | | | | | | | | |
| 1 | non-randomised studies | serious^a^ | very serious^b^ | not serious | not serious | publication bias strongly suspected^b^ | 37 | 37 | MD **1.26 higher** (4.51 higher to 1.99 higher) | | ⨁◯◯◯ Very low^a,b^ | | CRITICAL |
| **Before and Afte i-PRF + Arthrocentesis (follow-up: range 3 months to 6 months)** | | | | | | | | | | | | | |
| 7 | randomised trials | not serious | not serious | not serious | serious^c^ | none | 182 | 182 | MD **8.41 higher** (9.62 higher to 7.19 higher) | | ⨁⨁⨁◯ Moderate^c^ | | CRITICAL |
| **Before and After i-PRF + Arthroscopy (follow-up: range 3 months to 6 months)** | | | | | | | | | | | | | |
| 1 | non-randomised studies | serious^d^ | very serious^e^ | not serious | not serious | publication bias strongly suspected | 17 | 17 | MD **13.8 more** (18.39 more to 9.21 more) | | ⨁◯◯◯ Very low^d,e^ | | CRITICAL |
| **Arthrocentesis versus i-PRF + Arthrocentesis (follow-up: range 3 months to 6 months)** | | | | | | | | | | | | | |
| 7 | randomised trials | not serious | not serious | not serious | serious^f^ | none | 196 | 195 | MD **3.5 more** (4.69 more to 2.3 more) | | ⨁⨁⨁◯ Moderate^f^ | | CRITICAL |
| **Before and After i-PRF (follow-up: range 8 months to 12 months)** | | | | | | | | | | | | | |
| 1 | non-randomised studies | serious^a^ | very serious^b^ | not serious | not serious | publication bias strongly suspected | 37 | 37 | MD **7.29 more** (10.45 more to 4.13 more) | | ⨁◯◯◯ Very low^a,b^ | | CRITICAL |
| **Before and After i-PRF + Arthrocentesis (follow-up: range 8 months to 12 months)** | | | | | | | | | | | | | |
| 7 | randomised trials | not serious | not serious | not serious | serious^c^ | none | 143 | 143 | MD **8.99 more** (10.14 more to 7.84 more) | | ⨁⨁⨁◯ Moderate^c^ | | CRITICAL |
| **Before and After i-PRF + Arthroscopy (follow-up: range 8 months to 12 months)** | | | | | | | | | | | | | |
| 1 | non-randomised studies | serious^d^ | very serious^e^ | not serious | not serious | publication bias strongly suspected | 17 | 17 | MD **16 more** (20.49 more to 11.51 more) | | ⨁◯◯◯ Very low^d,e^ | | CRITICAL |
| **Arthrocentesis versus i-PRF + Arthrocentesis (follow-up: range 8 months to 12 months)** | | | | | | | | | | | | | |
| 1 | non-randomised studies | serious^g^ | not serious | not serious | not serious | none | 17 | 16 | MD **1.49 more** (0.88 more to 2.1 more) | | ⨁⨁⨁◯ Moderate^g^ | | CRITICAL |

**olute
(95% CI)**

**CI:** confidence interval; **MD:** mean difference

Explanations

a. According to the ROBINS-I tool, the Albilia study may have potential for confounding the effect of the intervention due to the absence of a control group. Based on the results, the author split the participants into two groups: those who responded to the treatment and continued receiving i-PRF injections, and non-responders, for whom the i-PRF injections were discontinued.

b. Since this group includes only one study (the Albilia study), there is a higher chance of inconsistency and a significant risk of publication bias.

c. The total sample size in the MMO analysis for the 3–6-month follow-up is 182 participants (ranging from 7 to 63 participants), while the 8-12 months follow-up includes a total of 143 participants (ranging from 7 to 63 participants). Most sample sizes in the "Before and After i-PRF + Arthrocentesis" group are small, which may lead to serious imprecision.

d. According to the ROBINS-I tool, in the González study, the start of follow-up and the start of the intervention do not coincide for most participants, and it is not clear whether any adjustment techniques were used to correct for selection biases.

e. Since this group includes only one study (the González study), there is a higher chance of inconsistency and a significant risk of publication bias.

f. The sample size is small, which could lead to serious imprecision.

g. The Yuce study presents a serious risk of bias due to an unbalanced comparison between the experimental groups. The i-PRF group received multiple injections, while the Hyaluronic Acid group received only a single injection, which may affect the study’s results. Additionally, the start of follow-up and the start of intervention do not coincide with most participants, as the procedures were carried out between 2015 and 2018. It is also unclear whether the outcome assessors were aware of the interventions received by the participants.

| **Supplementary Table 5.** PRISMA Checklist | | | |
| --- | --- | --- | --- |
| **Section and Topic** | **Item #** | **Checklist item** | **Location where item is reported** |
| **TITLE** | | |  |
| Title | 1 | Identify the report as a systematic review. | 1 |
| **ABSTRACT** | | |  |
| Abstract | 2 | See the PRISMA 2020 for Abstracts checklist. | 2 |
| **INTRODUCTION** | | |  |
| Rationale | 3 | Describe the rationale for the review in the context of existing knowledge. | 3,4 |
| Objectives | 4 | Provide an explicit statement of the objective(s) or question(s) the review addresses. | 4 |
| **METHODS** | | |  |
| Eligibility criteria | 5 | Specify the inclusion and exclusion criteria for the review and how studies were grouped for the syntheses. | 4,5 |
| Information sources | 6 | Specify all databases, registers, websites, organisations, reference lists and other sources searched or consulted to identify studies. Specify the date when each source was last searched or consulted. | 16 |
| Search strategy | 7 | Present the full search strategies for all databases, registers and websites, including any filters and limits used. | 5 |
| Selection process | 8 | Specify the methods used to decide whether a study met the inclusion criteria of the review, including how many reviewers screened each record and each report retrieved, whether they worked independently, and if applicable, details of automation tools used in the process. | 5 |
| Data collection process | 9 | Specify the methods used to collect data from reports, including how many reviewers collected data from each report, whether they worked independently, any processes for obtaining or confirming data from study investigators, and if applicable, details of automation tools used in the process. | 5 |
| Data items | 10a | List and define all outcomes for which data were sought. Specify whether all results that were compatible with each outcome domain in each study were sought (e.g. for all measures, time points, analyses), and if not, the methods used to decide which results to collect. | 5,6 |
|  | 10b | List and define all other variables for which data were sought (e.g. participant and intervention characteristics, funding sources). Describe any assumptions made about any missing or unclear information. | 5,6 |
| Study risk of bias assessment | 11 | Specify the methods used to assess risk of bias in the included studies, including details of the tool(s) used, how many reviewers assessed each study and whether they worked independently, and if applicable, details of automation tools used in the process. | 6 |
| Effect measures | 12 | Specify for each outcome the effect measure(s) (e.g. risk ratio, mean difference) used in the synthesis or presentation of results. | 6 |
| Synthesis methods | 13a | Describe the processes used to decide which studies were eligible for each synthesis (e.g. tabulating the study intervention characteristics and comparing against the planned groups for each synthesis (item #5)). | 4,5 |
|  | 13b | Describe any methods required to prepare the data for presentation or synthesis, such as handling of missing summary statistics, or data conversions. | 4-6 |
|  | 13c | Describe any methods used to tabulate or visually display results of individual studies and syntheses. | 5,6 |
|  | 13d | Describe any methods used to synthesize results and provide a rationale for the choice(s). If meta-analysis was performed, describe the model(s), method(s) to identify the presence and extent of statistical heterogeneity, and software package(s) used. | 6 |
|  | 13e | Describe any methods used to explore possible causes of heterogeneity among study results (e.g. subgroup analysis, meta-regression). | 6 |
|  | 13f | Describe any sensitivity analyses conducted to assess robustness of the synthesized results. | 6 |
| Reporting bias assessment | 14 | Describe any methods used to assess risk of bias due to missing results in a synthesis (arising from reporting biases). | 6 |
| Certainty assessment | 15 | Describe any methods used to assess certainty (or confidence) in the body of evidence for an outcome. | 6 |
| **RESULTS** | | |  |
| Study selection | 16a | Describe the results of the search and selection process, from the number of records identified in the search to the number of studies included in the review, ideally using a flow diagram. | 6 |
|  | 16b | Cite studies that might appear to meet the inclusion criteria, but which were excluded, and explain why they were excluded. | 6,7 |
| Study characteristics | 17 | Cite each included study and present its characteristics. | 6,7 |
| Risk of bias in studies | 18 | Present assessments of risk of bias for each included study. | 7 |
| Results of individual studies | 19 | For all outcomes, present, for each study: (a) summary statistics for each group (where appropriate) and (b) an effect estimate and its precision (e.g. confidence/credible interval), ideally using structured tables or plots. | 8 |
| Results of syntheses | 20a | For each synthesis, briefly summarise the characteristics and risk of bias among contributing studies. | 7 |
|  | 20b | Present results of all statistical syntheses conducted. If meta-analysis was done, present for each the summary estimate and its precision (e.g. confidence/credible interval) and measures of statistical heterogeneity. If comparing groups, describe the direction of the effect. | 7,8 |
|  | 20c | Present results of all investigations of possible causes of heterogeneity among study results. | 7 |
|  | 20d | Present results of all sensitivity analyses conducted to assess the robustness of the synthesized results. | 7 |
| Reporting biases | 21 | Present assessments of risk of bias due to missing results (arising from reporting biases) for each synthesis assessed. | 7 |
| Certainty of evidence | 22 | Present assessments of certainty (or confidence) in the body of evidence for each outcome assessed. | 7,8 |
| **DISCUSSION** | | |  |
| Discussion | 23a | Provide a general interpretation of the results in the context of other evidence. | 8-12 |
|  | 23b | Discuss any limitations of the evidence included in the review. | 11, 12 |
|  | 23c | Discuss any limitations of the review processes used. | 11,12 |
|  | 23d | Discuss implications of the results for practice, policy, and future research. | 11,12 |
| **OTHER INFORMATION** | | |  |
| Registration and protocol | 24a | Provide registration information for the review, including register name and registration number, or state that the review was not registered. | 4 |
|  | 24b | Indicate where the review protocol can be accessed, or state that a protocol was not prepared. | 4 |
|  | 24c | Describe and explain any amendments to information provided at registration or in the protocol. | 4,5 |
| Support | 25 | Describe sources of financial or non-financial support for the review, and the role of the funders or sponsors in the review. | 1 |
| Competing interests | 26 | Declare any competing interests of review authors. | 1 |
| Availability of data, code and other materials | 27 | Report which of the following are publicly available and where they can be found: template data collection forms; data extracted from included studies; data used for all analyses; analytic code; any other materials used in the review. | - |
